# Redox state affects fecundity and insecticide susceptibility in *Anopheles gambiae*

**DOI:** 10.1038/s41598-018-31360-2

**Published:** 2018-08-29

**Authors:** Cody J. Champion, Jiannong Xu

**Affiliations:** Department of Biology, New Mexico State University, Las Cruces, USA

## Abstract

Redox reactions play a central role in the metabolism of an organism. It is vital to maintain redox homeostasis in response to the fluctuation of redox shift in various biological contexts. NADPH-dependent reducing capacity is one of the key factors contributing to the redox homeostasis. To understand the redox capacity and its impact on mosquito fecundity and susceptibility to insecticides in *Anopheles gambiae*, we examined the dynamics of elevated oxidative state via induction by paraquat (PQ) and the inhibition of NADPH regeneration by 6-aminonicotinamide (6AN). In naïve conditions, inherent oxidative capacity varies between individuals, as measured by GSSG/GSH ratio. The high GSSG/GSH ratio was negatively correlated with fecundity. Both PQ and 6AN feeding increased GSSG/GSH ratio and elevated protein carbonylation, a marker of oxidative damage. Both pro-oxidants lowered egg production. Co-feeding the pro-oxidants with antioxidant lycopene attenuated the adverse effects on fecundity, implying that oxidative stress was the cause of this phenotype. Pre-feeding with 6AN increased insecticide susceptibility in DDT resistant mosquitoes. These results indicate that oxidative state is delicate in mosquitoes, manipulation of NADPH pool may adversely affect fecundity and insecticide detoxification capacity. This knowledge can be exploited to develop novel vector control strategies targeting fecundity and insecticide resistance.

## Introduction

*Anopheles gambiae* is the primary malaria vector in sub-Saharan Africa^1^. Vector competence is influenced by many parameters, including fecundity dependent population dynamics such as adult longevity and insecticide resistance^2–6^. Vectorial capacity relevant physiological systems, such as flight energetics, blood digestion, egg production and insecticide detoxification, are intertwined with various oxidation-reduction (redox) reactions^7–10^. If an excess amount of reactive oxygen species (ROS) is present in the cellular environment, oxidative damage to cellular materials occurs^11–13^. Defense against ROS damage utilizes several systems such as thioredoxin reductase, catalase, Cu-Zn SOD, antioxidants and glutathione systems^14^. NADPH is at the nexus of these systems, via providing reducing power needed by thioredoxin and glutathione systems or protection of catalase from oxidative and the resulting loss of function^15^.

Studies have demonstrated the link between oxidative stress and longevity, fecundity, and insecticide resistance in mosquitoes. In a permethrin resistant strain of *An. gambiae*, higher mitochondrial ROS production was coupled with decreased longevity in the insecticide-free condition, indicating a fitness cost of maintaining resistance associated detoxification capacity^16,17^. It has been shown that inhibiting oxidative defense enzymes, such as Cu-Zn SOD, GST, and catalase, resulted in increased sensitivity to insecticides in *An. arabiensis* and *An. funestus*^17^, indicating that oxidative defense is a critical component of insecticide resistance. DeJong *et al*. have shown that ROS detoxification via catalase is crucial for fecundity by protecting the embryo from damage in *An. gambiae*^18^. These studies indicate that oxidative stress defense at the direct enzymatic level, i.e., catalase, SOD, is essential to fecundity, longevity, and insecticide resistance. These redox reactions require sufficient reducing equivalent NADPH to operate^15^. However, little is known about how significant the NADPH dependent reducing power is to these defense mechanisms. NADPH is primarily produced through the oxidative phase of the pentose phosphate pathway (PPP)^19,20^. In this paper, we present the evidence that exposure to an oxidative stressor and metabolic inhibition of NADPH regeneration result in a redox shift, which leads to adverse effects on mosquito fecundity and insecticide detoxification. The vulnerability of NADPH dependent redox homeostasis presents a potential target for vector control.

## Results

### Pentose phosphate pathway is active during blood digestion

**Figure 1:**
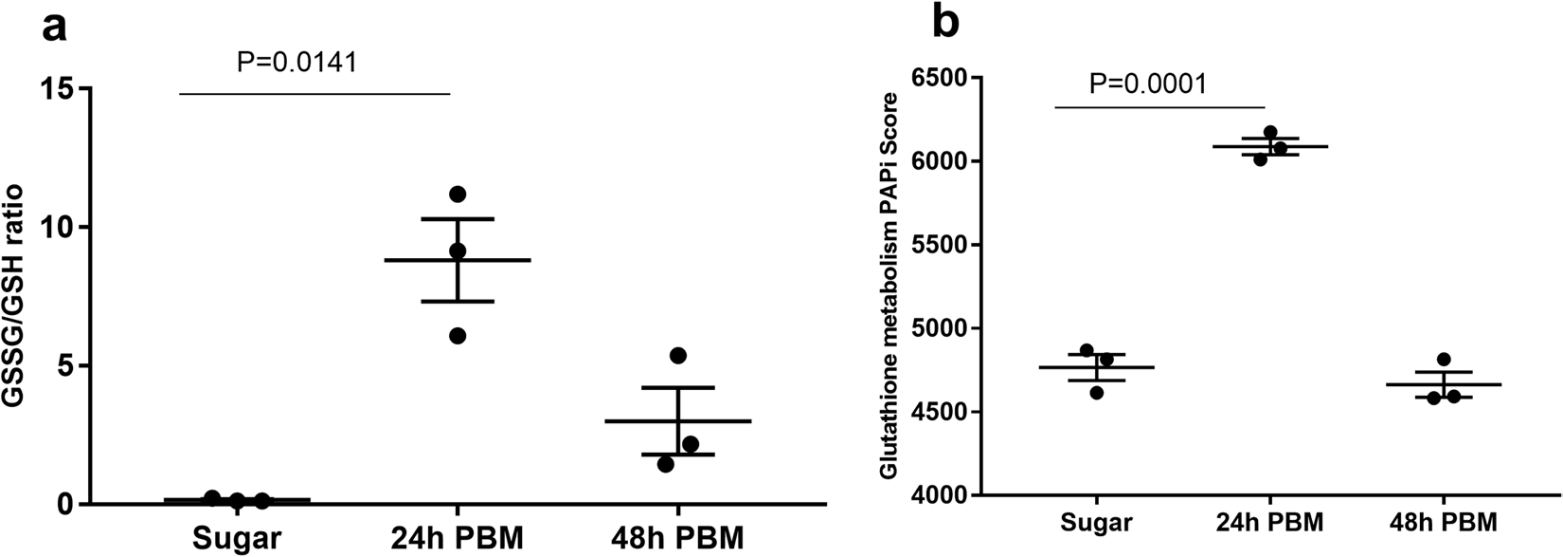
Glutathione metabolism is responsive to blood feeding. (**a**) GSSG/GSH ratios were measured from blood-fed females 24 h and 48 h PBM. Bars denote the mean with s.e.m. GSSG/GSH ratio is significantly increased at 24 h PBM (Kruskal-Wallis, P = 0.0036, Dunn’s test, P = 0.0141). (**b**) Pathway activity score for glutathione metabolism is increased significantly at 24 h PBM (One-way ANOVA, P < 0.001, Dunnett’s test, P = 0.0001). Bars denote mean with s.e.m.

**Figure 2:**
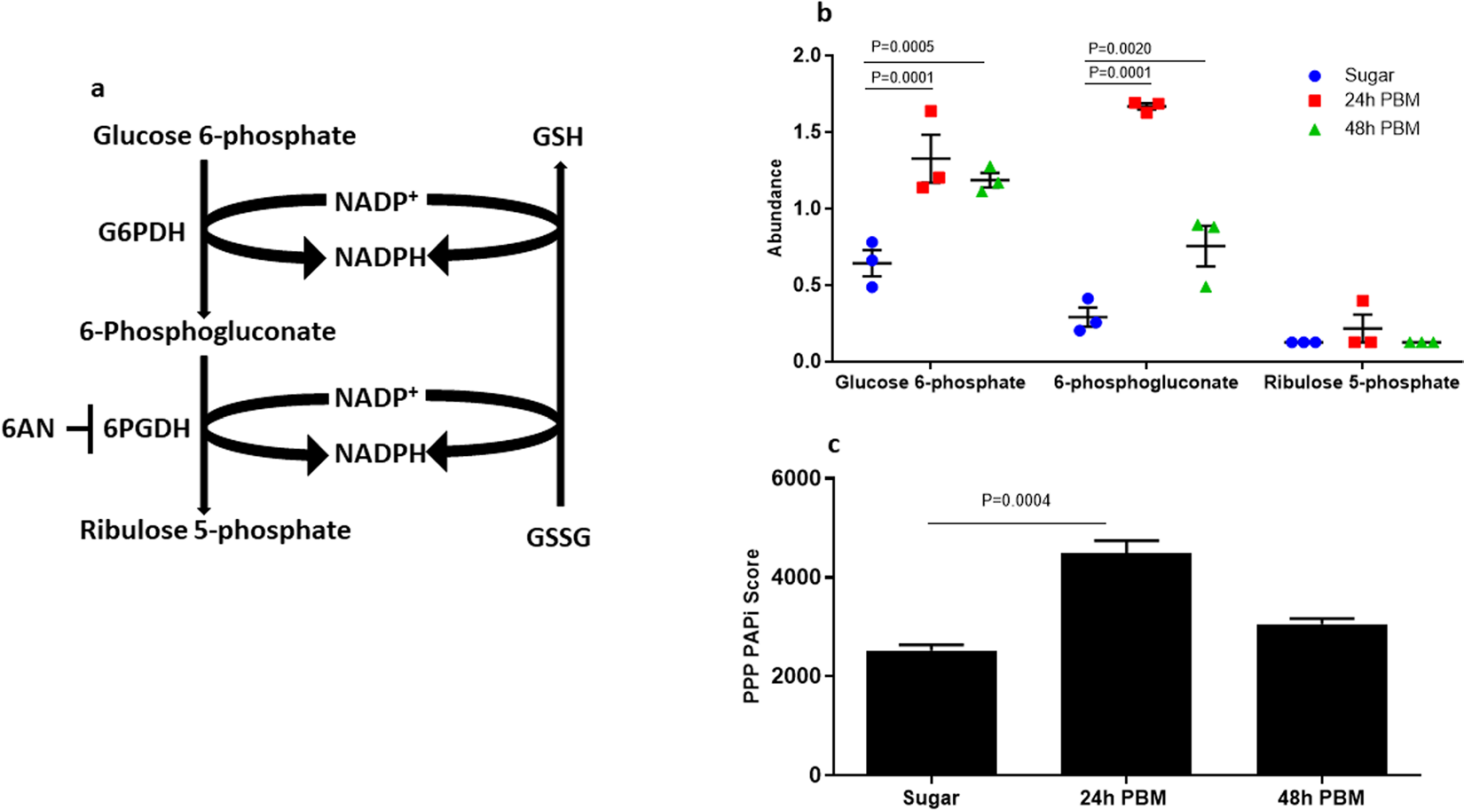
Pentose phosphate pathway associated metabolites and pathway activity are enriched after a blood meal. (**a**) A schematic representation of the major components of the PPP. (**b**) Compounds glucose 6-phosphate (Two-way ANOVA, time, biochemical and interaction terms were significant  P< 0.0001, Dunnett’s test with sugar as control, 24 h PBM P = 0.0001, and 48 h PBM P = 0.0005) and 6-phosphogluconate (Dunnett’s test with sugar as control, 24 h PBM,  P = 0.0001, and 48 h PBM, P = 0.0020) are at a higher abundance in blood-fed females both 24 h PBM and 48 h PBM. Symbols represent different cohorts with the central bar at the mean and error bars representing s.e.m. (**c**) PAPi activity score for the PPP. At 24 h PBM, the PPP activity is significantly increased (One-way ANOVA P = 0.0006, Dunnett’s test, P = 0.004). Error bars denote s.e.m.

**Figure 3:**
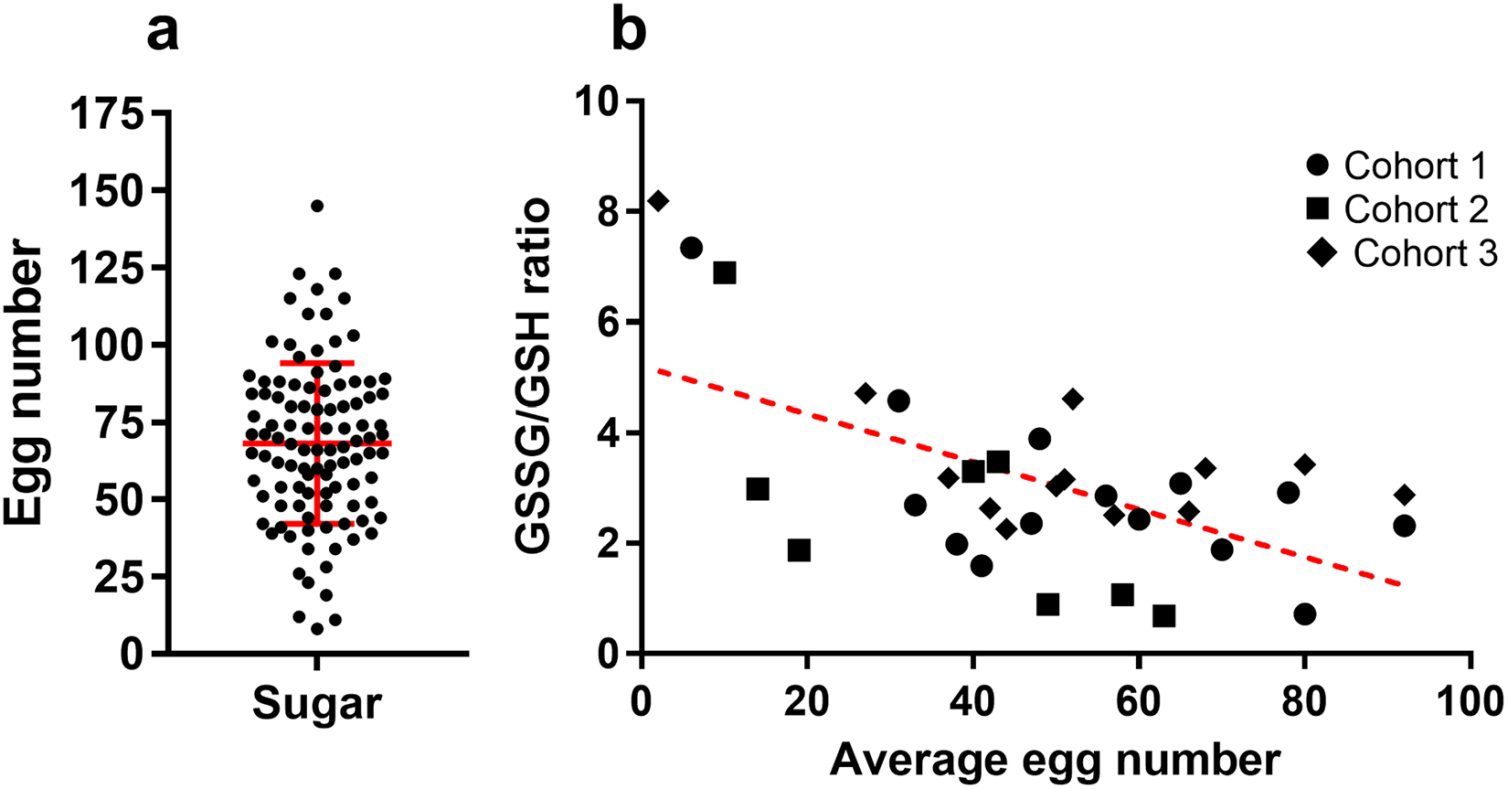
Egg number is highly variable in the mosquito cohort and is inversely correlated with the GSSG/GSH ratio. (**a**) Distribution of individual egg counts in the G3 population (n = 104). The center line denotes the mean with standard deviation bar. (**b**) A negative correlation between the GSSG/GSH ratio and egg counts was observed. The red dotted line shows the best fit line. Females were blood fed after three days post-emergence and ovaries were dissected 3 days after the blood feeding for egg counting. The carcasses without ovaries were used for GSSG and GSH quantitation.

Previously, we conducted a metabolomics survey of the midgut with sugar- and blood-fed conditions^21^. We examined the GSH and GSSG abundance in the midgut metabolome at 24 h and 48 h post blood meal (PBM). GSSG/GSH ratio was increased at 24 h post blood meal compared to the sugar-fed condition (Fig. 1a). The metabolic fluxes of glutathione metabolism were increased at 24 h post blood meal (Fig. 1b), as revealed by PAPi analysis^22^. The fluxes of glutathione metabolism and elevated GSSG/GSH ratio post blood feeding suggest that redox status fluctuates in the midgut environment during the blood digestion. Reducing power in the form of NADPH is vital to maintaining redox balance in many cellular processes such as growth, metabolism, and detoxification^23,24^. PPP is a significant source for the oxidative generation of NADPH^20,25^. This system is composed of glucose-6-phosphate dehydrogenase (G6PDH) and 6-phosphogluconate dehydrogenase (6-PGDH) which act on glucose 6-phosphate and 6-phosphogluconate, respectively, to reduce NADPH from NADP^+^ (Fig. 2a). In the midgut metabolome profiles^21^, some substrates associated with this pathway (glucose 6 phosphate and 6-phosphogluconate) are increased 24 h PBM (Fig. 2b). Similarly, pathway activity score is increased at the same time point (Fig. 3c). These data suggest that the PPP is active in the midgut microcosm during blood digestion, which likely contributes to the redox homeostasis via NADPH regeneration. It is noteworthy that the midgut metabolites may be derived from multiple sources including host midgut cells, mammalian blood cells and gut-resident microbial cells, based on the experimental setting of the metabolomic study^21^. Overall, the metabolomic profiles suggest that metabolic activities in the midgut act for maintaining redox homeostasis after a blood meal is taken.

### Fecundity is negatively correlated with elevated oxidative status

There is an inherent variation in respect of fecundity. In a single mosquito cohort, different individuals may produce vastly different numbers of eggs. As shown in Fig. 3a, the egg counts varied in a range of 0–143/female, with a mean ± SD of 68 ± 25.9. To determine the relationship between fecundity and oxidative status, the GSSG/GSH ratio was examined in female mosquitoes and the correlation between egg number and GSSG/GSH ratio was determined by using the Spearman correlation test. The data were generated from three cohorts; the egg number per female was 0–95. Ovaries were examined for egg number, and carcasses of three females who produced similar egg numbers were pooled for quantifying GSSG/GSH ratio. As shown in Fig. 3b, the GSSG/GSH ratio was inversely correlated with the egg number (Spearman’s r = −0.03837, two-tail, P = 0.0229). These data suggest that individuals differ in their redox state and elevated oxidative state adversely affects egg production.

### Mosquito survivorship is sensitive to oxidative stress imposed by paraquat feeding

**Figure 4:**
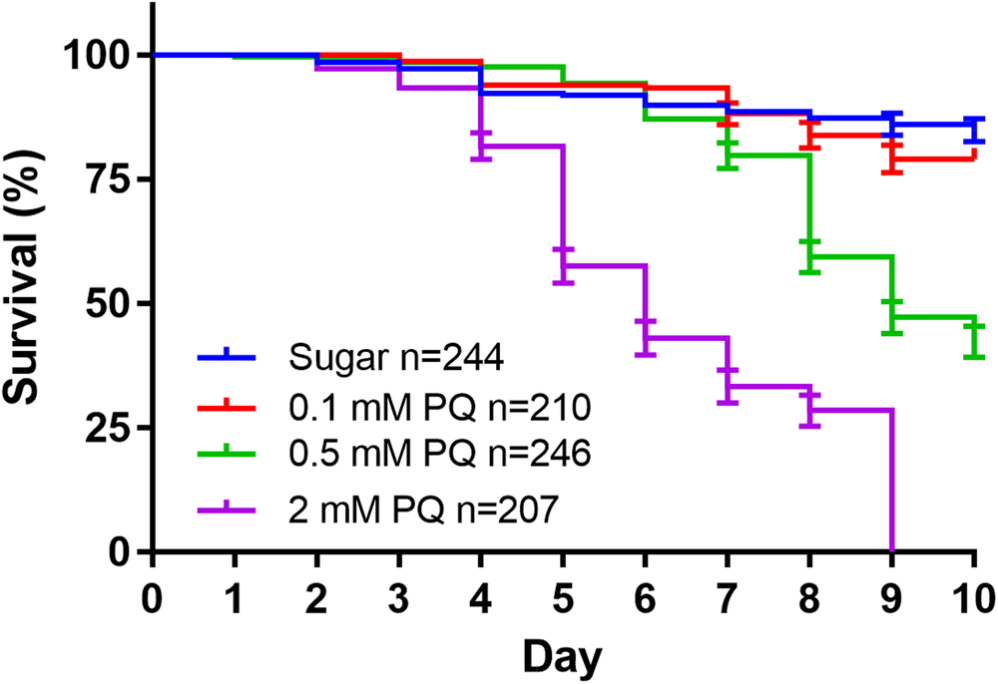
Survival curves of PQ fed female mosquitoes. Each curve represents data obtained from 4 to 5 cohorts, with standard error bars. Higher lethality was caused by 0.5 mM and 2 mM PQ (Mantel-Cox, P < 0.0001), but not 0.1 mM PQ (P > 0.05).

Next, we tested the mosquito sensitivity to oxidative stress induced by an oxidative stressor, paraquat (PQ). PQ undergoes redox cycling *in vivo* through several mechanisms utilizing NADPH as an electron donor^26,27^. PQ has been used as an oxidative stress inducer in insects^28^. Mosquitoes were exposed to PQ that was orally administrated via sugar meals. Fig. 4 shows a dose-dependent mortality curve in a 10-day experimental period, a dose of 2 mM PQ caused a higher death rate at day 5 post-exposure (Mantel-Cox, P < 0.0001) and 0.5 mM PQ induced significant death rate at day 8 post-exposure (Mantel-Cox, P < 0.0001). The mosquitoes were tolerant to 0.1 mM PQ; the mortality was not different from that of the naïve control (Mantel-Cox, P > 0.05). The 0.1 mM PQ was chosen for further experiments. A 6AN dose-dependent experiment was also performed, no lethal dose of 6AN was found (data not shown). Therefore, 10 mM 6AN was used for further experiments.

### Induced oxidative stress reduces fecundity

**Figure 5:**
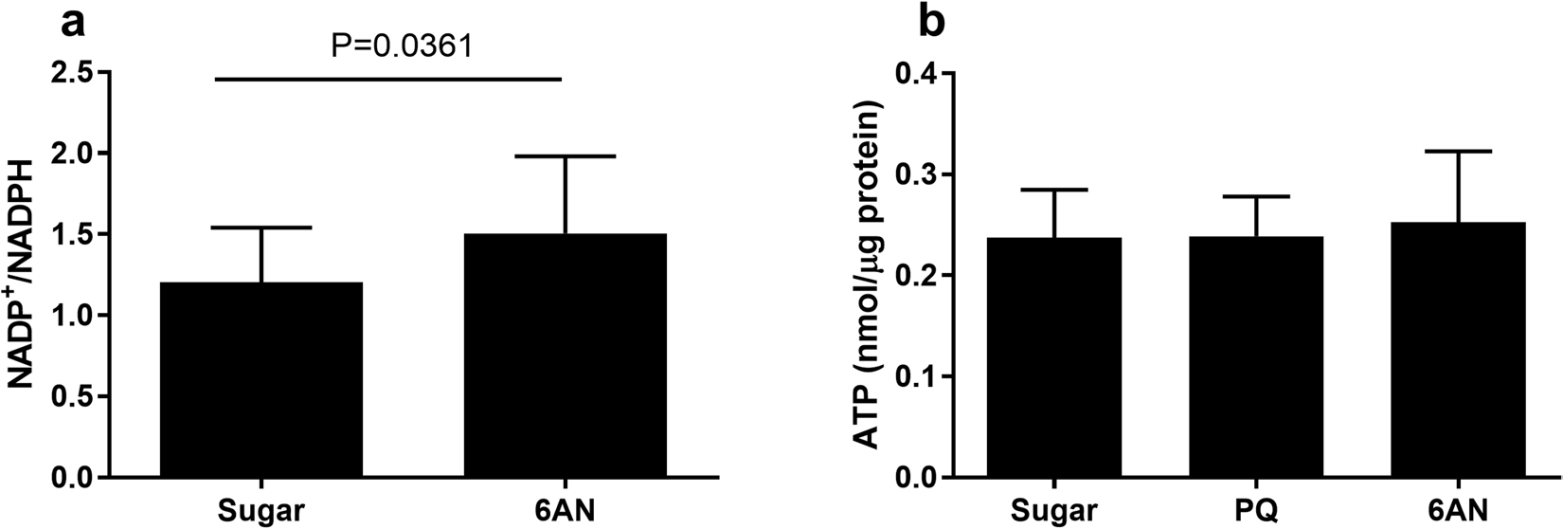
6AN and PQ target and off-target validation. (**a**) NADP^+^/NADPH ratio was elevated in 10 mM 6AN fed mosquitoes (Mann-Whitney, P = 0.0361). (**b**) ATP production was not changed between 10% sucrose fed mosquitoes and 0.1 mM PQ or 10 mM 6AN mosquitoes (One-way ANOVA, P = 0.7926). Error bars denote the standard deviation.

6AN is a well-established inhibitor of 6-phosphogluconate dehydrogenase (6-PGDH)^29,30^, which selectively inhibits 6-PGDH 400-fold more efficiently than other enzymes associated with NADPH regeneration^31^. To verify the action of 6AN on NADPH generation in mosquitoes, NADP^+^/NADPH ratio was determined in mosquitoes that were fed on 10 mM 6AN for 5 days. As shown in Fig. 5a, the ratio was elevated in 6AN-fed mosquitoes compared to that in sugar-fed control (Mann-Whitney test, P < 0.05), indicating that 6AN treatment reduced NADPH regeneration as expected. To determine if energy balance is potentially impacted by either 6AN or PQ, ATP concentration in 10 mM 6AN and 0.1 mM PQ fed groups was measured. The ATP quantity was 0.24 ± 0.05, 0.24 ± 0.04, and 0.25 ± 0.07 nmol/µg protein in sugar-fed, PQ-fed and 6AN-fed cohorts, respectively (Fig. 5b), suggesting that the treatments did not affect ATP production.

**Figure 6:**
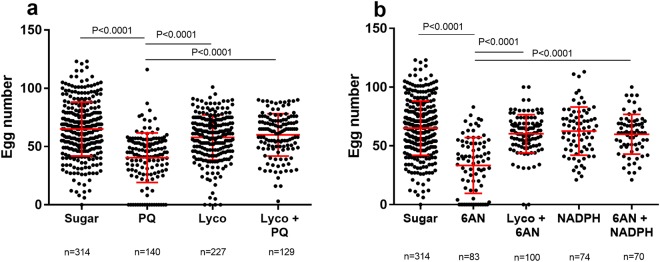
Decreased egg number from PQ and 6AN stressed mosquitoes. Each point represents one female. Center bar represents the mean with standard deviation bar. The same sugar control group is used in both panels PQ (**a**) and 6AN (**b**). Oxidative stress induction reduced egg number (10 mM 6AN One-way ANOVA, P < 0.0001, Dunn’s test, P < 0.0001 and 0.1 mM PQ Kruskal-Wallis, P < 0.0001, Dunn’s test, P < 0.0001). Fecundity reduction in both 10 mM 6AN and 0.1 mM PQ groups was reversed when the antioxidant lycopene was added (0.1 mM PQ + 10 mM Lyco vs. 0.1 mM PQ, Dunn’s test, P < 0.0001, and 10 mM 6AN + 10 mM Lyco vs. 10 mM 6AN, Dunn’s test, P < 0.0001). Fecundity reduction in the 10 mM 6AN group was reversed when NADPH was co-applied (10 mM 6AN + 10 mM NADPH vs. 10 mM 6AN, Dunn’s test, P < 0.0001).

Thereafter, the effect of PQ and 6AN on fecundity was determined. Mosquitoes were treated with 0.1 mM PQ for 5 days before a blood meal was given for egg production. The persistent exposure to low dose PQ imposed a chronic pressure on oxidative balance, which had an adverse effect on mosquito fecundity. Stressed mosquitoes produced fewer eggs (Dunn’s test, P < 0.0001). This detrimental effect was reversed when the mosquitoes were co-fed PQ with lycopene (Lyco), a potent antioxidant (Fig. 6a). Similarly, when mosquitoes were treated with 10 mM 6AN to repress the generation of reducing agent NADPH, egg production dropped significantly (Dunn’s test, P < 0.0001). A reversal of the lower fecundity was achieved by co-feeding 6AN with lycopene or NADPH leading to normal egg production (Fig. 6b). At least three cohorts were used for each treatment and all data were pooled for comparison.

### PQ and 6AN elevate oxidative stress

**Figure 7:**
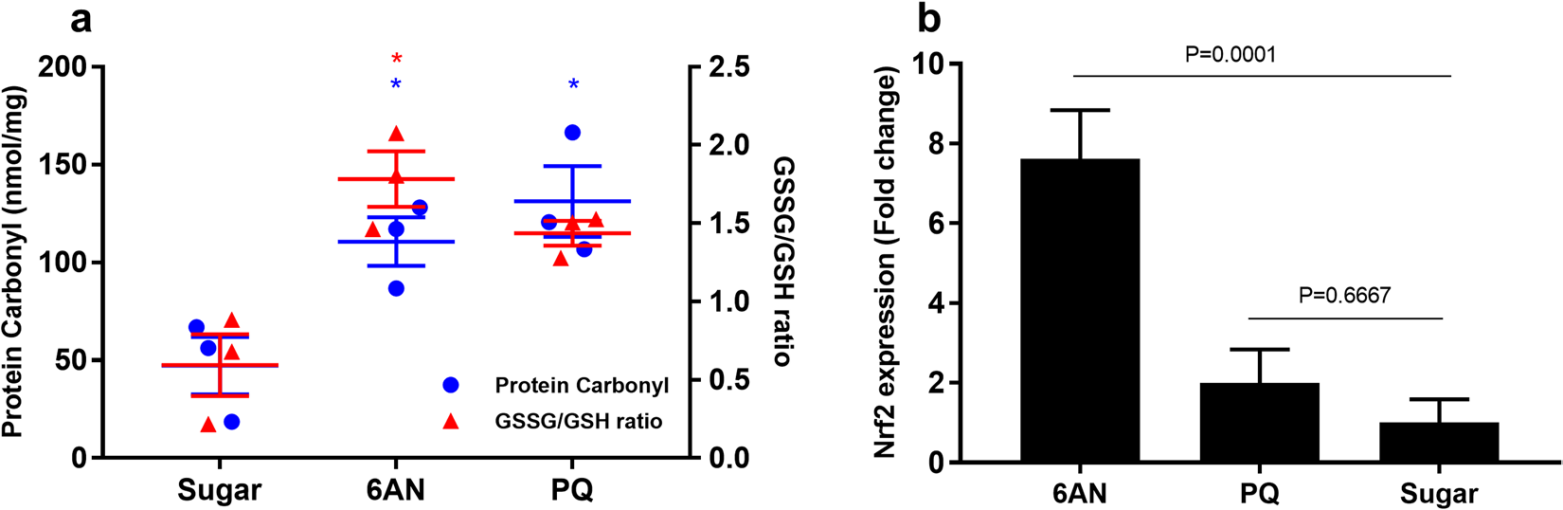
Increased oxidative stress was induced by 10 mM 6AN or 0.1 mM PQ. The oxidative marker GSSG were increased at 24 h post blood meal. (**a**) Protein carbonylation and GSSG/GSH ratios. Each symbol represents data from a pool of 30 females; the central line denotes the mean with a standard error bar. Statistically significant comparisons are denoted with an asterisk. GSSG/GSH ratio in 10 mM 6AN was significantly increased (One-Way ANOVA, P < 0.0001, Dunnet’s test, P = 0.0341). Protein carbonylation was increased significantly in 10 mM 6AN, and 0.1 mM PQ groups (One-Way ANOVA, P < 0.0001, Dunnett’s test, P = 0.0446 and 0.0141, respectively). (**b**) Gene *Nrf2* expression pattern of two separate cohorts. Transcription of *Nrf2* was elevated in 10 mM 6AN groups only (One-way ANOVA, p < 0.0001, Dunnett’s test, p < 0.0001). Error bars represent the s.e.m.

To determine the redox status in the mosquitoes treated with PQ or 6AN, two biomarkers of oxidative stress were measured: GSSG/GSH ratios and protein carbonylation, a marker of oxidative damage. Mosquitoes were fed with either sugar, 10 mM 6AN or 0.1 mM PQ for 5 days before a blood meal was given. The mosquitoes were processed for the two assays at 24 h post blood feeding. In control mosquitoes, the GSSG/GSH ratio was less than 1, while in PQ and 6AN fed mosquitoes, the ratio was greater than 1.5 (Fig. 7a), and the ratio was significantly higher in 6AN groups (Dunnett’s test, P = 0.0341). Similarly, protein carbonylation was significantly increased in both 6AN and PQ fed groups (Dunnett’s test, P = 0.0446 and 0.0141, respectively). These data indicate that both PQ and 6AN induced oxidative stress in mosquitoes.

Nuclear factor erythroid 2 (NFE2)-related factor 2 (Nrf2) is a transcription factor mediating a variety of antioxidant defense mechanisms^32^. A qPCR experiment was conducted to examine the transcription of *Nrf2* in response to the treatments. In stressed mosquitoes, the expression of *Nrf2* was significantly upregulated in 6AN groups when compared to the expression in control mosquitoes (Dunnett’s test, P = 0.0001), and a trend of upregulation was observed in PQ groups (P = 0.6667) (Fig. 7b).

### Stressed mosquitoes are more susceptible to insecticides

**Figure 8:**
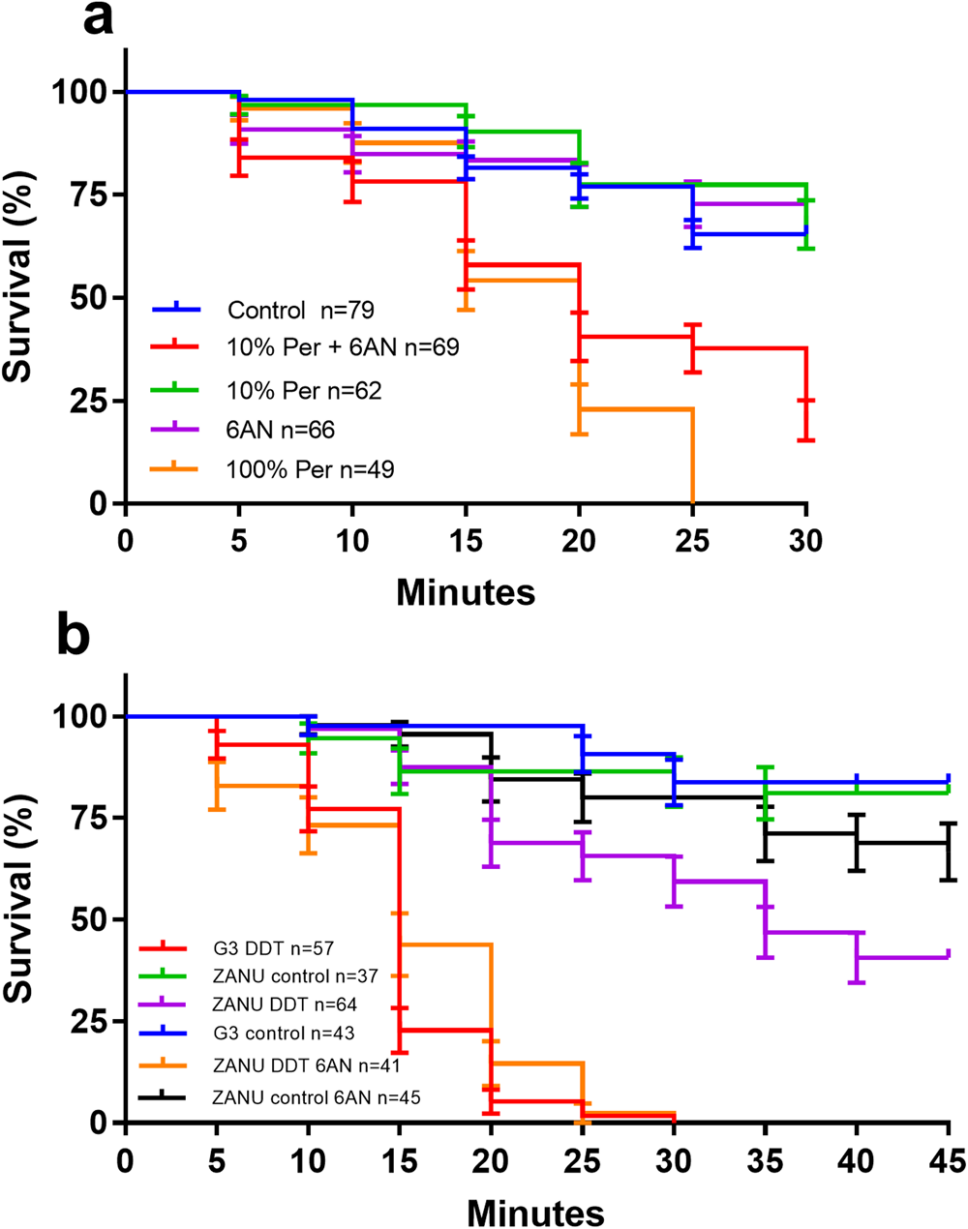
Susceptibility to permethrin and DDT was increased in mosquitoes when pre-fed with 6AN. (**a**) In a permethrin susceptibility test, the G3 mosquitoes are susceptible to the diagnostic dose of permethrin (Per) (21.5 µg permethrin/bottle) (Mantel-Cox, P < 0.0001), and tolerant to the 10% of the diagnostic dose of Per (2.15 µg permethrin/bottle). Pre-feeding with 10 mM 6AN sensitized the mosquitoes to the 10% of the diagnostic dose of Per (Mantel-Cox, P < 0.0001). (**b**) In a DDT susceptibility test, the ZAN/U strain regained insecticide susceptibility when pre-fed with 10 mM 6AN (Mantel-Cox, P < 0.0001). Data were pooled from at least two separate experiments.

Next, the effect of oxidative status on insecticide susceptibility was investigated in the permethrin susceptible G3 strain and the DDT resistant, ZAN/U strain^33^. G3 strain was sensitive to permethrin. As shown in Fig. 8, the mortality was 100% at 25 min post-exposure to permethrin at a diagnostic dose (2.15 µg permethrin/bottle), while the mortality was 30% at 25 min post-exposure to solvent acetone only. When the mosquitoes were exposed to a lower dose, i.e., 10% of the diagnostic dose of permethrin (0.215 µg permethrin/bottle), the survival curve was similar to that of the mosquitoes exposed to acetone control (Mantel-Cox, P > 0.05). Mosquitoes appear tolerant to this dose. However, after mosquitoes were pre-fed with 10 mM 6AN to reduce NADPH dependent reducing power and then exposed to the lower dose of permethrin, the survival was significantly lower (Mantel-Cox, P < 0.0001). The 6AN pre-feeding had a negligible effect on survival (Fig. 8a). Inhibiting NADPH regeneration with 6AN increased the susceptibility to permethrin. In the DDT resistant ZAN/U strain, a similar trend is observed (Fig. 8b). The G3 survival at 30 min post-DDT exposure was 0, while the ZAN/U survival was 59%. The survival of G3 and ZAN/U to acetone control was 83% and 81%, respectively. Pre-feeding with 6AN resensitized the ZAN/U mosquitoes to DDT; all 6AN treated mosquitoes died at 30 minutes of DDT exposure (Mantel-Cox, P < 0.0001).

## Discussion

Maintenance of redox homeostasis in insects has been shown to be critical for fecundity^34–36^, longevity^17,37^, insecticide resistance^17^, and immunity^38–40^. In mosquitoes, ROS production is responsible for defense against infection^39,41–44^. ROS detoxification is vital to control the oxidative damage after blood feeding. Antioxidant enzymes Cu Zn and Mn superoxide dismutase (SOD), catalase, and glutathione peroxides and thioredoxin reductase protect cells from free radical damage, as reviewed by Graca-Souza *et al*.^10^. However, knowledge of the metabolic configurations behind the ROS detoxification and redox homeostasis in mosquitoes remains limited. In this study, we investigated the effect of redox manipulation on mosquito fecundity and insecticide detoxification in mosquitoes.

First, we examined the metabolic profiles^21^ in the midgut metabolome data during blood digestion, which demonstrates that glutathione metabolism and PPP are active in the midgut (Figs 1 and 2). It is noteworthy that the metabolites in the midgut metabolome may be derived from mosquito host, mammal blood, and resident microbes. During the evolution, mosquitoes host has evolved various strategies to cope with redox fluctuation associated with blood digestion^10^. Consequentially, anti-oxidant agents are elevated post blood feeding, as demonstrated in the metabolome data^21^, such as increased urate level, a well-known oxidant scavenger^45^, increased cysteine level and reduction of cystine/cysteine ratio, which likely resulted from low cysteine oxidation, in addition to the active glutathione metabolism and PPP, a major source of reducing agent NADPH. These metabolic profiles suggest that, in the gut microcosm, the net metabolic outcome from all involved parties makes the redox towards a reduction state during the blood digestion, as shown in *Aedes aegypti*^46^. Such adaptation ensures the blood-meal dependent reproduction is successfully achieved^10^.

However, this genetic ability varies individually in a population. The individual variation in egg production (Fig. 3a) is negatively correlated with elevated oxidative stress (Fig. 3b), suggesting that the inherent differences in oxidative stress defense capability may contribute to the variability in fecundity between individuals. Our finding is in line with the report by DeJong *et al*. that catalase isoforms at codon 2 (Ser2Trp) have different efficiencies in enzyme functions, which underlines the fecundity difference in two mosquito strains each carries a different isoform^18^. These data imply that global oxidative status and fecundity are inherently intertwined. We hypothesize that the redox homeostasis behind blood digestion and egg production requires sufficient reducing agent in the form of NADPH. To test this hypothesis, we manipulated mosquito redox status by two means. First, a chronic level of oxidative stress was induced by feeding PQ at a sublethal concentration 0.1 mM (Fig. 4); and second, NADPH regeneration was reduced by feeding 10 mM 6AN to inhibit pentose phosphate pathway (Fig. 5a). ATP is unaffected by 6AN or PQ feeding (Fig. 5b), suggesting both treatments did not affect energy balance significantly. In both contexts, the treatments induced adverse effects on fecundity, which was reversed by administration of the anti-oxidant, lycopene or NADPH (Fig. 6a,b). When redox balance was disturbed, oxidative stress levels were elevated, and oxidative damage occurred, demonstrated by elevated GSSG/GHS ratio and protein carbonylation (Fig. 7a). The Nrf2 signaling mediates a defense mechanism against oxidative stress^47^. Recently, Nrf2-mediated signaling was shown to play roles in redox biology in *Aedes aegypti*, affecting embryo survival, midgut redox homeostasis, xenobiotic metabolism and vectoral adaptation^48^. In our study, 6AN significantly and PQ marginally upregulated the transcription of *Nrf2* (Fig. 7b), suggesting that the Nrf2 mediated defense was triggered to cope with the systemic state of oxidative stress in the contexts. These observations indicate that the observed phenotypes in the 6AN treatments are due to lowered NADPH metabolism, and the phenotypes in the PQ treated groups are caused by direct oxidative damage as well as NADPH depletion by PQ. These effects are unlikely to be an off-target effect such as reducing cellular energy by interfering with NADH involved reactions, in the case of 6AN treatment, or by damage to the mitochondria, in the case of PQ treatment. However, we would like to point out that our data were generated from pharmacological interventions, it is possible that unrecognized off-target effects may contribute to the phenotypes. Further studies using a different approach to manipulate PPP function are needed to support these conclusions.

Insecticide resistance has developed in *An. gambiae* populations via selective pressure from both long-lasting insecticide-treated bed nets (LLIN), indoor residual, and agricultural spraying^49–53^. Several metabolic detoxification mechanisms have been shown to be responsible for insecticide resistance, such as overproduction of esterases, cytochrome P450 monooxygenases (P450) system, and glutathione-S-transferases (GST)^54–57^. Cytochrome P450 system mediated insecticide detoxification requires NADPH cytochrome P450 reductase (CPR), which transfers electrons from NADPH to oxidation reactions^58^. Therefore, sufficient supply of reducing equivalent NADPH is critical for the metabolic resistance via the P450 system. We hypothesize that blocking NADPH regeneration by 6AN will impair detoxification capacity, therefore, increase the susceptibility to insecticides. Indeed, pre-feeding with 6AN made susceptible G3 strain more sensitive to permethrin (Fig. 8a), and the 6AN treatment resensitized the DDT resistant strain ZAN/U to DDT (Fig. 8b). This is likely due to the reduction of NADPH levels that could have been used for NADPH dependent CPR. The DDT resistance of ZAN/U strain is mediated by metabolic detoxification. Recent evidence has shown that *CPR* gene is required for metabolic resistance in bed bugs and locusts^59–61^. In addition, Oliver and Brooke have demonstrated that oxidative stressors introduced by dietary copper sulfate and hydrogen peroxide induced acute stress and impaired the capacity to detoxify insecticides^17^. These data emphasize the connection between insecticide metabolism and redox state.

In summary, this work provides a line of evidence that NADPH metabolism is supportive of redox homeostasis, which is critical for mosquito fecundity and xenobiotic detoxification. NADPH provides reducing equivalents for various oxidation-reduction reactions involving in reductive biosynthesis as well as cellular defense system against oxidative stress^15,62^. Regeneration of GSH from GSSG by glutathione reductase requires electrons donated by NADPH. Catalases are bound by NADPH, which keeps catalases active for disposing of hydrogen peroxide^63^. In addition, as an electron carrier, NADPH is a critical component of the thioredoxin system, which is essential for redox regulation of protein function and signaling via thiol redox control^64^. Regeneration of NADPH from NADP^+^ largely depends on the pentose phosphate pathway, although nicotinamide nucleotide transhydrogenase (NNT), cytosolic isocitrate dehydrogenase (IDH), and cytosolic malic enzyme (MEN) are other enzymatic players as well^65^. Interestingly, *6-PGDH* and *NNT* genes are bloodmeal inducible, while *IDH* and *MEN* genes are not^66^, suggesting the NADPH demand is high after blood feeding. This network coordinates metabolic responses to various environmental stress, such as oxidative stress, starvation, and desiccation. To ensure proper functionality of various NADPH dependent cellular functions and mitigation of ROS toxicity, it is crucial to maintaining a sufficient supply of NADPH. As demonstrated in this study, the shortage of NADPH derived from inhibiting 6-PGDH in the PPP impairs mosquito fecundity and resensitizes resistant mosquitoes to insecticides. Accordingly, we propose that suppression of the NADPH pool will interfere with multiple processes that are critical for fecundity, longevity, and insecticide resistance. This will be a novel target for developing innovative vector control measures.

## Methods

### Mosquito strains

*Anopheles gambiae* G3 strain was reared under standard conditions (28 °C ± 1 °C, 70–80% RH, 12-hour light-dark cycle) as described previously^67^. Mosquitoes were maintained on 10% sucrose solution changed daily, and outbred mice were given as a blood source for egg production. *An. gambiae* DDT resistant strain, ZAN/U (MRA-594)^33^ obtained through BEI Resources, NIAID. Selection was performed every 5 generations using 0.4 mg/L DDT exposure for 24 hours at the L4 stage. Rearing condition was identical to the G3 strain.

### Correlation of egg number and GSSG/GSH ratio

Females were fed 10% sucrose for three days, and then blood fed. Three days post blood feeding eggs were counted, and females were grouped in groups of 3 based on egg number.

### Egg numeration

Ovaries were dissected at day 3 post blood feeding. The number of eggs per female was numerated as a measure of fecundity.

### NADP^+^/NADPH and ATP quantification

In these assays, cohorts of mosquitoes were fed with 10 mM 6AN in 10% sucrose for five days. To prepare samples for the assays, 30 females from a cohort were homogenized in 300 µl of 1× PBS. This was done with 10 cohorts in the ATP assay and 13 cohorts in the NADP^+^/NADPH assay. This homogenate was centrifuged for 1 min at 13,000 × g, and the supernatant was decanted and filtered through a 10 kDa cutoff centrifuge filter (13,000 × g for 20 min). The supernatant was then used in the NADP^+^/NADPH quantification assay (Catalog number MAK038, Sigma-Aldrich) or ATP quantification assay (Catalog number MAK190, Sigma-Aldrich) per the manufacturer’s instruction.

### GSSG/GSH quantification

To determine if there is a correlation between variation of egg production and oxidative status, GSSG and GSH were quantified in carcass. The ovary dissection was carried out to numerate eggs at 3 day post blood feeding, carcasses of the mosquitoes were pooled into groups of 3 (data presented in Fig. 3) or 30 (data presented in Fig. 7), homogenized in 15 µl of 1× PBS per carcass, and 25% 5-Sulfosalicylic Acid (SSA) was added to the sample to create a solution with 5% SSA. Then, the samples were centrifuged at 8,000 g for 10 min at 4 °C. The reduced (GSH) and oxidized (GSSG) glutathione was quantified using a GSSG/GSH quantification kit (Catalog number G257-10, Dojindo Molecular Technologies, Inc.) following the manufacturer’s instruction. Total glutathione and GSSG were determined, respectively, and then GSH was calculated by calculating the difference between the total glutathione and GSSG. To quantify GSSG and GSH in stressed mosquitoes, newly emerged mosquitoes were treated with 0.1 mM PQ or 10 mM 6AN for 5 days, then fed on blood to induce egg production. At day 3 post blood feeding, ovaries were dissected for egg counting, and carcasses were used for GSSG and GSH quantitation as described above.

### Protein carbonylation

Mosquitoes were treated with 0.1 mM PQ or 10 mM 6AN for 5 days, then fed on blood to induce egg production. At day 3 post blood feeding, after ovary dissection, the carcasses of mosquitoes were pooled into groups of 30, homogenized in 15 µl of 1× PBS per carcass. The samples were centrifuged at 10,000 g for 10 min at 4 °C. The protein carbonylation and total protein were then quantified using a protein carbonylation kit (Item number 10005020, Cayman chemical) following manufacturer’s instruction.

### Paraquat, 6AN and lycopene treatment

Oxidative stress inducer, Paraquat (N,N′-dimethyl-4,4′-bipyridinium dichloride) was purchased from Sigma-Aldrich. PQ in different concentrations (0.1–2 mM) was provided in 10% sucrose solution to mosquitoes. PQ-treated mosquitoes were subject to different measures as designed. 6AN is an inhibitor of 6-PGDH, which was purchased from Cayman chemical company. 6AN (10 mM) was provided to mosquitoes in 10% sucrose for 5 days. Due to low solubility, 6AN was dissolved at 37 °C and then mixed with 10% sucrose. Lycopene is a potent antioxidant. Redivivo lycopene was a gift provided by DSM Food Specialties B.V. 10 mM lycopene in 10% sucrose solution was given to mosquitoes for 5 days.

### CDC bioassay

Mosquito susceptibility to permethrin (Sigma-Aldrich) was determined using the CDC bottle assay^68^. Sugar fed *An. gambiae* G3 or ZAN/U females that had been fed 10 mM 6AN in 10% sucrose for three days were used. The mosquitoes were exposed to the CDC diagnostic dose (100% permethrin (21.5 µg/bottle)) or 10% of the diagnostic dose (2.15 µg/bottle). Mosquitoes that were unable to fly and unresponsive were counted every 5 minutes for 30 mins. DDT (Dichlorodiphenyltrichloroethane) (100 µg/bottle) was used for ZAN/U resistance testing. Diagnostic time for DDT treated bottles was 45 minutes.

### RT-PCR

Three separate cages were fed 0.1 mM PQ, 10 mM 6AN, or 10% sucrose for 5 days. Females were starved of sugar for 8 hours and then blood fed. Ten females from each cohort were dissected at 24 h post blood meal to remove midgut. RNA was extracted from mosquito carcass using TRIzol (Invitrogen). cDNA was synthesized using cDNA Reverse Transcription Kit (New England Biolabs). Quantitative PCR was performed using a CFX Connect Real-Time System (BIO RAD) using Power SYBR-green PCR master MIX (ThermoFisher Scientific). *Rps7* was used as an endogenous control. The primer sequences are: Nrf2-F, 5′-TCA CCG TAC GCA TTT CTT GGT-3′; Nrf2-R, 5′-GCT GAC GTT CAT GGC ATT CTG-3′; Rps7-F, 5′-GCG TGA GGT CGA GTT CAA CA-3′; Rps7-R, 5′-GGG AAC GCG GTC TCT TCT-3′. The amplification program was 95 °C for 3 min; then the following sequence repeated 39 times; 95 °C for 10 sec followed by 60 °C for 30 seconds. Once these steps were completed, a 95 °C step followed by a 65 °C for 5 sec step was done to complete the program. The normalized expression (ΔΔCq) of target gene *Nrf2* was presented as the relative quantity of *Nrf2* normalized to the quantity of reference gene *Rps7* in samples, which was implemented using the software in the CFX Connect Real-Time System.

### Pathway activity profiling (PAPi) analysis

Pathway Activity Profiling (PAPi)^69^, a network algorithm, was used to quantify metabolic pathway activities using metabolite abundance and known metabolic pathways. These pathways were retrieved from KEGG. R version 3.3.3 and package PAPi was used to calculate the pathway activity score. The metabolic dataset was generated and described in a dataset paper previously^21^ and is available from the Dryad repository: https://datadryad.org/resource/doi:10.5061/dryad.88r38.

### Statistical tests

All statistical tests, unless otherwise noted, were performed using GraphPad Prism version 7.00 for Windows, GraphPad Software, La Jolla California USA. Significance was defined for all tests as α = 0.05.

#### Survival analysis

Survival statistics were calculated using the Kaplan-Meier method to calculate survival fractions. Then survival curves were compared using the Log-rank (Mantel-Cox) test using pre-planned pairs, i.e., sugar feding death rate vs. 0.1 mM PQ feeding death rate.

#### ATP quantification

The ATP quantities were compared between sugar-, PQ- and 6AN-treated mosquitoes. After checking for normality (Shapiro-Wilks, p > 0.05), a standard one-way ANOVA was performed.

#### NADPH/NADP^+^ quantification

The NADPH/NADP^+^ ratio was compared between 6AN-treated mosquitoes and sugar-fed controls. A Mann-Whitney one-tailed test was performed.

#### Fecundity

To compare effects of different treatments on fecundity, egg numbers of each group were analyzed using multivariate techniques. The data were pooled from all replicates and tested for equal variance via Barlett’s test (α = 0.05). If the data did significantly have different variances then the Kruskal-Wallis test, the non-parametric method, was used. If the Kruskal-Wallis test showed significant differences between treatment (α = 0.05) level, then Dunn’s multiple comparisons were used for further pairwise comparisons.

#### GSSG/GSH ratio

Since GSSG and GSH taken as a ratio from the same pool as total glutathione, non-parametric techniques were used in this analysis. These measurements were taken from three biological replicates. A Kruskal-Wallis test was then used to determine if the difference between treatment levels were present (α = 0.05). If significant then Dunn’s multiple comparison tests were used to compare each treatment to the sugar-fed control.

#### GSSG/GSH and egg number correlation

A non-parametric correlation test (Spearman test), was used to determine the relationship between GSSG/GSH and egg number. Both r and one-tailed P-value were reported.

#### RT-PCR

RT-PCR data were analyzed via CFX Manager Software (Bio-Rad). Expression levels were calculated using sugar-fed females as a control sample. Data were generated from two cohorts, and a one-way ANOVA was performed followed by Dunnett’s multiple comparisons using sugar-fed as the control.

#### Biochemical abundance comparison in PAPi analysis

Biochemical compound abundance at sugar-fed, 24 h and 48 h post blood feeding was compared. A one-way ANOVA followed by Dunnett’s multiple comparisons using sugar-fed as control was used.

## Data Availability

All data are available from authors upon request.
